# Improving Comfort of Patients with Ureteral Obstruction and Malignant Disease Should Be of Concern

**DOI:** 10.1089/jpm.2016.0276

**Published:** 2016-11-01

**Authors:** Benoît Vogt, François-Noël Desfemmes, Arnaud Desgrippes

**Affiliations:** Department of Urologie, Polyclinique de Blois, La Chaussée Saint-Victor, France.

Dear Editor:

The ureteral stents are defined as devices that are inserted inside obstructive ureters to maintain patency. Ureteral obstruction caused by extrinsic compression is a challenge in the management of malignant diseases.^1,2^

First, adequate stent placement across an obstructed ureter does not necessarily guarantee urine patency.^1^ Moreover, the stent is poorly tolerated, severely impairing the quality of life of patients, and general tolerance remains unchanged with time.^3^ These symptoms are due largely to the bladder irritation caused by the stent and the reflux in the kidney. Thus, the stent can induce additional suffering to the patient.

Obstruction or pain associated with ureteral stent is significant and is–or should be–of concern.

Since 2010, we are trying to improve the patient's quality of life, especially in malignant ureteral obstruction. We developed a new stent, the JFil^®^, with a thread of suture as a means of decreasing urinary symptoms.^4^ But, this stent cannot be used for meatus obstruction in the bladder. We developed another stent to determine exactly the ureteral length of the patient and adjust the distal stent segment into the meatus. A latex end piece is attached at the bottom of the stent to prevent the stent from slipping off. We used this customized procedure in more than 40 cases and the first results are encouraging. The study design was approved by French Ethical Committee (CPP 2015-09-11).

We present the case of a 63-year-old patient in remission 20 years after rectal cancer treatment with surgery, chemotherapy, and radiotherapy.

**FIG. 1.**
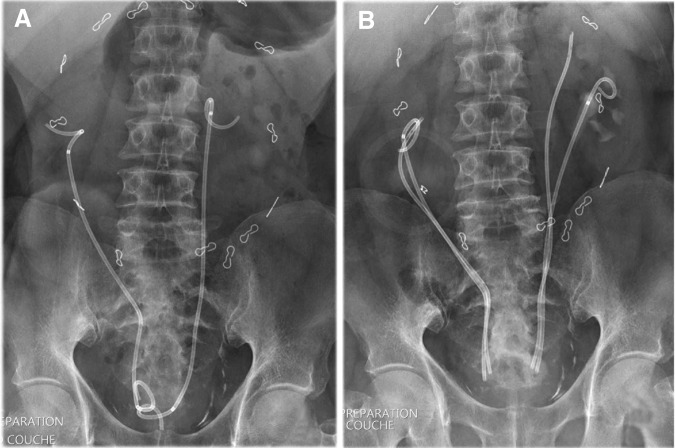
Appearance of ureteral stents on X-ray. **(A)** Double-pigtail tumor stent with obstruction and incontinence. **(B)** Sectioned tandem ureteral stents and disappearance of obstruction and incontinence.

Ureteral stents insertion was performed for fibrosis ureteral obstruction with renal failure (Fig. 1A). The patient has clearly described symptoms associated with these stents: urinary frequency, urgency, and incontinence.

After recurrent stent obstruction and acute pyelonephritis, stent replacement was attempted with an alternative option by tandem ureteral stents. This procedure, with four stents into the bladder, allowed to maintain kidney function but impaired the quality of life with severe incontinence.

We thought that, by decreasing the amount of material in the bladder, it may be possible to attenuate the symptoms of this patient.

With the section of the stents to the exact length of the ureters, incontinence disappeared (Fig. 1B). These stents were replaced twice a year and the patient's quality of life is much improved.

We have recently created a silicone stent's design more suited to the ureter with a nonrefluxing system and we will start a comparative study with the double-pigtail stent. Improving comfort of patients with ureteral obstruction and malignant disease is possible and should be a priority.
